# Intravenous abatacept in Japanese patients with polyarticular-course juvenile idiopathic arthritis: results from a phase III open-label study

**DOI:** 10.1186/s12969-019-0319-4

**Published:** 2019-04-30

**Authors:** Ryoki Hara, Hiroaki Umebayashi, Syuji Takei, Nami Okamoto, Naomi Iwata, Yuichi Yamasaki, Yasuo Nakagishi, Toshitaka Kizawa, Ichiro Kobayashi, Tomoyuki Imagawa, Noriko Kinjo, Norihito Amano, Yoko Takahashi, Masaaki Mori, Yasuhiko Itoh, Shumpei Yokota

**Affiliations:** 1 Department of Pediatrics, Yokohama City University School of Medicine, 3-9 Fukuura, Kanazawa-ku, Yokohama-shi, Kanagawa 236-0004 Japan; 2 Department of General Pediatrics, Miyagi Children’s Hospital, 4-3-17 Ochiai, Aoba-ku, Sendai-shi, Miyagi 989-3126 Japan; 3 Department of Pediatrics, Kagoshima University Medical and Dental Hospital, 8−35−1 Sakuragaoka, Kagoshima-shi, Kagoshima, 890-8544 Japan; 4 Department of Pediatrics, Graduate School of Medicine, Osaka Medical College, 2-7 Daigaku-machi, Takatsuki-shi, Osaka, 569-8686 Japan; 5 Department of Immunology and Infectious Diseases, Aichi Children’s Health and Medical Center, 1-2 Osakata, Morioka-cho, Oobu-shi, Aichi 474-8710 Japan; 6 Department of Pediatric Rheumatology, Hyogo Prefectural Kobe Children’s Hospital, 1-6-7 Minamimachi, Minatojima, Chuo-ku, Kobe-shi, Hyogo 650-0047 Japan; 7 Department of Pediatrics, Japan Community Health Care Organization Sapporo Hokushin Hospital, 1-2, 2-jo, 6-chrome, Atsubetsu-chuo, Atsubetsu-ku, Sapporo-shi, Hokkaido 004-8618 Japan; 8 Department of Pediatrics, Hokkaido University Hospital, North-15, West-7, Sapporo-shi, Hokkaido 060-8638 Japan; 9 Division of Infection, Immunology and Rheumatology, Kanagawa Prefectural Hospital Organisation Kanagawa Children’s Medical Center, 2-138-4 Mutsukawa, Minami-ku, Yokohama-shi, Kanagawa 232-8555 Japan; 10 Department of Pediatrics, University of the Ryukyus Hospital, 207 Aza Uehara, Nishihara-cho, Nakagami-gun, Okinawa, 903-0215 Japan; 11 Bristol-Myers Squibb K.K, 6-5-1 Nishi-Shinjuku, Shinjuku-ku, Tokyo, 163-1328 Japan; 12 Department of Lifetime Clinical Immunology, Graduate School of Medical and Dental Sciences, Tokyo Medical and Dental University, 1-5-45 Yushima, Bunkyo-ku, Tokyo, 113-8510 Japan; 13 Department of Pediatrics, Graduate School of Medicine, Nippon Medical School, 1-1-5 Sendagi, Bunkyo-ku, Tokyo, 113-8603 Japan; 14 Fuji Toranomon Orthopaedics Hospital, 1067-1 Kawashimata, Gotenba-shi, Shizuoka, 412-0045 Japan

**Keywords:** Abatacept, Disease-modifying anti-rheumatic drugs (DMARDs), Japanese, Juvenile idiopathic arthritis, Pharmacokinetics

## Abstract

**Background:**

To investigate efficacy and safety of intravenous abatacept in Japanese patients with active polyarticular-course juvenile idiopathic arthritis (pJIA).

**Methods:**

In this phase III, open-label, multicenter, single-arm study, patients with pJIA aged 4–17 years who failed ≥1 biologic or methotrexate received weight-tiered (< 75 kg: 10 mg/kg; 75–100 kg: 750 mg; > 100 kg: 1000 mg) intravenous abatacept at Weeks 0, 2, 4, and every 4 weeks thereafter. The study comprised a short-term period (16 weeks) and ongoing long-term period. Primary endpoint: Week 16 JIA-American College of Rheumatology criteria 30 (JIA-ACR30) response rate. Secondary endpoints/outcomes included Week 16 JIA-ACR50/70/90 response and inactive disease rates, Childhood Health Assessment Questionnaire-Disability Index (CHAQ-DI), pharmacokinetics, safety, and immunogenicity. Proportions of patients achieving Juvenile Arthritis Disease Activity Score in 27 joints using C-reactive protein (JADAS27-CRP) remission (score < 1) and minimal disease activity (MDA; score < 3.8), were among exploratory endpoints.

**Results:**

All 20 patients who received study medication completed the short-term period. During the long-term period, two patients discontinued due to insufficient efficacy or patient decision. Median age and disease duration at baseline were 10.5 and 0.75 years, respectively. Week 16 JIA-ACR30 response rate (primary endpoint) was 90.0% (18/20). JIA-ACR50/70/90 response and inactive disease rates at Week 16 were 75.0% (15/20), 70.0% (14/20), 35.0% (7/20), and 25.0% (5/20), respectively. At Week 52, JIA-ACR30/50/70/90 response and inactive disease rates were observed by 88.9% (16/18), 88.9% (16/18), 83.3% (15/18), 66.7% (12/18) and 44.4% (8/18), respectively. CHAQ-DI improved after Week 12. JADAS27-CRP remission and MDA were achieved by 15.0% (3/20) and 45.0% (9/20) of patients at Week 16, and by 50.0% (9/18) and 78.0% (14/18) of patients at Week 52, respectively. The mean abatacept pre-dose serum concentration was above the target therapeutic exposure (10 μg/ml) from Week 8 through Week 16. All adverse events were of mild/moderate intensity, except for one case of severe gastroenteritis. No deaths, malignancies, or autoimmune disorders were observed. No antidrug antibodies were detected through Week 16; one patient had a positive immunogenic response during the cumulative period.

**Conclusion:**

Intravenous abatacept was efficacious and well tolerated in Japanese patients with active pJIA.

**Trial registration:**

ClinicalTrials.gov: NCT01835470. Date of registration: April 19, 2013.

## Background

Juvenile idiopathic arthritis (JIA) is a pediatric rheumatic disease of unknown etiology that presents in children before the age of 16 years [1]. In developed countries, JIA occurs with a prevalence of 16–150 cases per 100,000 children [1], with the estimated prevalence in Japan being 10–15 cases per 100,000 children [2]. Polyarticular-course JIA (pJIA) is the most common JIA subtype [1, 2], and is defined as the presence of ≥5 affected joints within the first 6 months of onset of disease symptoms [3, 4].

In Japan, methotrexate (MTX) is the recommended first-line disease-modifying antirheumatic drug (DMARD) therapy for pJIA [2]. If disease activity remains moderate or high following 3 months of MTX treatment, second-line treatment should be initiated with biologic DMARDs, such as a tumor necrosis factor-α inhibitor (TNFi) or interleukin (IL)-6 receptor blocker [5, 6]. Although the TNFis etanercept and adalimumab and the IL-6 receptor blocker tocilizumab have been approved for the treatment of JIA in Japan [7–9], alternative treatment options still need to be investigated for patients who are intolerant or do not respond to available conventional synthetic and biologic DMARDs, or who lose response over time [10–18].

Abatacept is a recombinant fusion protein comprising the extracellular domain of human cytotoxic T-lymphocyte-associated protein-4 (CTLA4) linked to a modified human immunoglobulin (Ig) G1 Fc portion that selectively modulates the CD80/CD86:CD28 costimulatory signal that is required for full T-cell activation [19]. Compared with other currently available treatments for rheumatoid arthritis (RA), abatacept has a fundamentally different mechanism of action from other biologic DMARDs, as it targets the underlying process of T-cell activation [20, 21]. Abatacept has been shown to reduce disease progression, and improve function and health-related quality of life in RA [22–24]. The intravenous (IV) formulation of abatacept is effective and well tolerated in pJIA [12, 15, 25, 26], and has been approved for the treatment of active pJIA in patients over 2 years old in the USA [20] and over 6 years old in Canada [27] and Europe [28]. In Japan, IV abatacept is approved for the treatment of adult patients with RA and in February 2018, approval was granted for the treatment of pediatric patients with pJIA [29].

This study was designed to investigate the efficacy, pharmacokinetics (PK), safety, and immunogenicity of IV abatacept in Japanese patients with pJIA. Here, the 1-year interim results are presented. To our knowledge, this is the first publication of data for abatacept treatment in Japanese patients with pJIA.

## Patients and methods

### Study design

This single-arm, open-label, multicenter, two-part, phase III study (NCT01835470) was initiated in September 2013 and conducted across 13 centers in Japan. During the 16-week short-term period, patients received IV abatacept at Week 0 (Day 1), Week 2 (Day 15), Week 4 (Day 29), and then every 4 weeks (Q4W) at a dose based on the patient’s weight at each visit (< 75 kg: 10 mg/kg; 75–100 kg: 750 mg; > 100 kg: 1000 mg), with or without MTX (4–10 mg/m^2^/week). After completion of the short-term period, patients entered the long-term period, during which they continued to receive IV abatacept Q4W until approval of abatacept for the treatment of pJIA in Japan, or termination of abatacept development by the study sponsor (Bristol-Myers Squibb K.K.). Here we report efficacy and PK data for the short-term period (up to Week 16 [Day 113]) and cumulative efficacy and safety data for the short- and long-term periods combined; cumulative efficacy data are reported to study Week 52 (Day 365), and cumulative safety data are reported to study Week 136 (Day 953). The long-term period is ongoing for most patients.

During the short-term period, the use of MTX (4–10 mg/m^2^/week) or low-dose oral corticosteroids (≤10 mg/day or ≤ 0.2 mg/kg/day, whichever was less) and non-steroidal anti-inflammatory drugs was permitted at stable doses, although the dose of these agents could be reduced if an adverse event (AE) occurred and subsequently increased to the original starting dose if that AE resolved. During the cumulative period, the use of all conventional synthetic and biologic DMARDs was prohibited, with the exception of MTX. Patients who completed the long-term period or who discontinued abatacept treatment early during the short-term or long-term periods subsequently entered a follow-up period and were assessed at 4, 12, and 24 weeks after their last dose of study medication. Patients who started commercial abatacept were not permitted to enter the follow-up period.

### Patients

Eligible patients were Japanese, aged 4–17 years, and met the International League of Associations for Rheumatology criteria for JIA [3], with a diagnosis of one of the following: extended oligoarticular JIA, polyarthritis rheumatoid factor (RF) positive, polyarthritis RF negative, or systemic JIA with a polyarticular course and absence of systemic features within the 6 months prior to enrollment. Patients were required to have a history of ≥5 active joints with active disease and active articular disease at baseline, defined as ≥2 active joints and ≥ 2 joints with limitation of motion (LOM), and inadequate therapeutic response or intolerance to ≥1 biologic DMARD or MTX, as determined by the examining physician.

Patients were excluded if they had systemic onset JIA with any of the following manifestations within the 6 months prior to enrollment: intermittent fever due to JIA, rheumatoid rash, hepatosplenomegaly, pleuritis, pericarditis, or macrophage activation syndrome. Patients with another rheumatic disease or major chronic infectious, inflammatory or immunologic disease (e.g. psoriatic arthritis, inflammatory bowel disease, spondyloarthropathy, hypogammaglobulinemia, or systemic lupus erythematosus) were also excluded from the study.

### Study endpoints

The primary study endpoint was JIA-American College of Rheumatology (ACR) criteria 30% improvement (JIA-ACR30) response rate at Week 16. JIA-ACR30 was defined as a ≥ 30% improvement in at least three of the six JIA-ACR core set variables and a > 30% worsening in no more than one of the six JIA-ACR core set variables [30]. Inactive disease (modified criteria) was defined as no active joints, a Physician’s Global Assessment (PGA) score of ≤10 mm, and a C-reactive protein (CRP) value of ≤0.3 mg/dL [31]. Secondary study endpoints and outcomes were JIA-ACR50, 70, and 90 response rates [14], inactive disease rate at Week 16, and physical function measured using the Disability Index of the Childhood Health Assessment Questionnaire (CHAQ-DI) [32] at Week 16, as well as PK, safety and tolerability, and immunogenicity during the short-term period. Exploratory endpoints and outcomes included JIA-ACR30, 50, 70, and 90 response rates, inactive disease rate, juvenile arthritis disease activity score 27 active joint count-CRP (JADAS27-CRP) remission rate [33–36], long-term safety during the cumulative period, and immunogenicity during the cumulative period and 6 months following discontinuation of treatment. The Institute of Clinical Outcomes Research and Education (ICORE, Woodside, CA, USA) provided the licensing to use the CHAQ-DI in this study [32].

### Efficacy assessment

Efficacy assessments were performed before administration of study medication at each visit. The following six JIA-ACR core set variables were assessed [37]: number of active joints, number of joints with LOM, PGA of disease activity (scale: 0–100 mm), Parental Global Assessment of patient overall well-being (PaGA; scale: 0–100 mm), physical functional as measured by the CHAQ-DI (scale: 0–3; completed by the patient or their parent/caregiver), and laboratory measure of inflammation, as measured by serum CRP level.

JADAS27-CRP was calculated as the sum of the scores of the following four components: PGA of disease activity, PaGA of overall well-being, active joint count in 27 joints, and CRP level. Remission was defined as JADAS27-CRP < 1, and minimal disease activity was defined as JADAS27-CRP < 3.8.

### PK assessment

The serum abatacept concentration was determined using a sensitive and validated enzyme-linked immunosorbent assay (Covance Inc., Trenton, NJ, USA), using a biotinylated monoclonal mouse anti-human CTLA4 antibody (clone 11D4), as described previously [38]. The validated linear assay range was 1.0–30.0 ng/mL. The PK parameters assessed were pre-dose observed serum concentration (C_trough_) measured at Weeks 2, 4, 8, 12, and 16, and maximum observed serum concentration (C_max_) measured at Weeks 8, 12, and 16.

### Safety assessment

Safety was evaluated during the short-term, long-term, and follow-up study periods by monitoring of AEs and laboratory tests. All AEs were coded using the Medical Dictionary for Regulatory Activities version (MedDRA) 19.0. Causality assessment between each AE and study medication was performed by the investigator.

### Immunogenicity assessment

Serum samples were collected prior to administration of the study medication at Weeks 0, 8, and 16 during the short-term period, and Weeks 32 and 52, then every 6 months, during the long-term period. A validated sensitive electrochemiluminescence assay was used to detect and evaluate anti-drug antibodies (ADA) specific to ‘CTLA4 ± Ig’ and ‘Ig and/or junction region’ in serum samples [38, 39]. A sample was considered seropositive if immunodepletion was observed with abatacept or truncated CTLA4 and reported as positive with a titer of ≥10.

### Statistical analyses

There was no formal statistical hypothesis or testing for this study. All patients who received at least one dose of study medication were included in the efficacy, PK, safety, and immunogenicity analyses. For the JIA-ACR30, 50, 70, and 90 response rates, and inactive disease status analyses, two-sided 95% confidence intervals (CIs) were computed using an exact method based on the binomial distribution. For the short-term period efficacy analysis, any patient who prematurely discontinued the trial after receiving study medication had missing data imputed as an ACR non-response at all scheduled protocol visits up to Week 16 subsequent to the point of discontinuation. The cumulative period efficacy analysis was performed based on as-observed data. The proportion of JIA-ACR30 responders was summarized at Week 16 for the following subgroups: gender, baseline age, JIA subtype, concomitant MTX therapy, and prior biologic treatment. PK summary statistics of mean and standard deviation were presented for C_trough_ and C_max_ by day in the short-term period. AEs, serious AEs (SAEs), deaths, discontinuations due to AEs, clinical laboratory abnormalities, changes in vital signs, and positive immunogenicity rates were summarized.

### Sample size

The sample size of 20 patients was determined based on the operational feasibility of a local JIA study. In a preceding JIA study (NCT00095173), JIA-ACR30 response rate was 63.7%. Based on the assumption of a JIA-ACR30 response rate of 65%, a sample size of 20 treated patients would provide a two-sided exact 95% CI of 40.8 to 84.6%.

## Results

### Patients

**Fig. 1:**
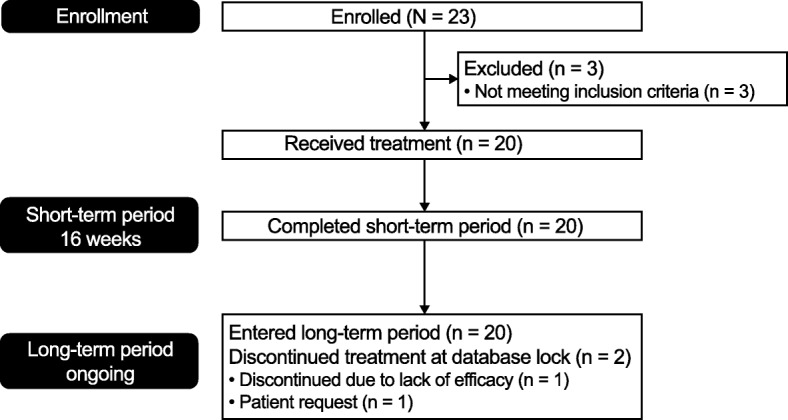
Patient disposition

A total of 23 patients were enrolled in the study, three of whom were excluded as they no longer met study criteria (Fig. 1). All 20 patients who received treatment completed the short-term period and subsequently entered the long-term period. At Week 52, 2/20 (10%) patients had discontinued treatment during the long-term period: one withdrew consent and did not enter the follow-up period, the other discontinued due to lack of efficacy and completed the follow-up period. Most patients were female, with a median baseline age of 10.5 years and disease duration of < 1 year (Table 1); at baseline, 80% of patients were receiving concomitant MTX and 20% had previously taken one or more biologic DMARD(s). Twelve of 20 patients (60%) from the overall population received > 24 infusions of abatacept over the cumulative period (i.e. to Week 52 from last enrolled patient’s first treatment).

**Table 1:** Baseline demographics and patient characteristics

Baseline characteristic	Patients (*N* = 20)
Age, years	10.5 (5–16)
Age group, n (%)	
4–8 years	8 (40)
9–12 years	7 (35)
13–17 years	5 (25)
Weight, kg	37.9 (15.4–68.3)
Female, n (%)	15 (75)
Race: Japanese, n (%)	20 (100)
Disease duration, years	0.75 (0.2–11.9)
Number of active joints	6.0 (2.0–19.0)
Number of joints with LOM	4.0 (2.0–10.0)
Parent Global Assessment, VAS 100 mm	37.5 (0–94.0)
Physician Global Assessment, VAS 100 mm	37.0 (10–80.0)
CHAQ-DI	0.63 (0–2.88)
CRP, mg/dL	0.58 (0.02–2.67)
JADAS27-CRP	12.0 (4.2–26.4)
JIA disease subtype at study entry, n (%)	
Extended oligoarticular	2 (10)
Polyarticular (RF negative)	8 (40)
Polyarticular (RF positive)	10 (50)
Systemic	0
Prior biologic therapy, n (%)	4 (20)
Tocilizumab	3 (15)
Anti-TNF therapy	4 (20)
Adalimumab	4 (20)
Etanercept	1 (5)
Concomitant MTX therapy at Day 1, n (%)	16 (80)
MTX dose, mg/m^2^/week	8.9 (6.0–10.3)

Data are median (minimum–maximum) unless specified otherwise

*CHAQ-DI* Childhood Health Assessment Questionnaire-Disability Index, *JADAS27-CRP* juvenile arthritis disease activity score 27 active joint count-C-reactive protein, *JIA* juvenile idiopathic arthritis, *LOM* limitation of motion, *MTX* methotrexate, *RF* rheumatoid factor, *SD* standard deviation, *TNF* tumor necrosis factor, *VAS* visual analog scale

### Efficacy

**Fig. 2:**
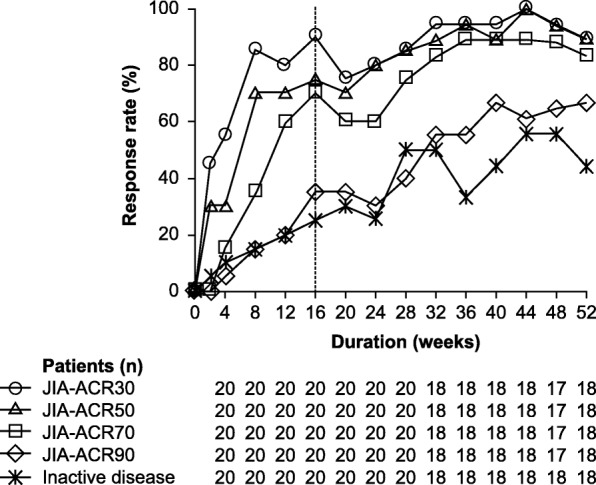
Time courses of JIA-ACR pediatric response rates and inactive disease rates from baseline to Week 52 of the cumulative period. The proportion of patients who achieved JIA-ACR30 (open circle), JIA-ACR50 (open triangle), JIA-ACR70 (open square), JIA-ACR90 (open diamond), and inactive disease (asterisk) was evaluated at indicated time points (all treated patients; *N* = 20). Data from Week 2 through Week 16 were analyzed with non-responder imputation (patients with missing data were considered as non-responders). Data from Week 20 through Week 52 were analyzed using observed cases (only patients who were in the study at the time point being evaluated). *JIA-ACR30/50/70/90* Juvenile idiopathic arthritis-American College of Rheumatology criteria 30/50/70/90% improvement

The proportion of patients who achieved JIA-ACR30, 50, 70 and 90 response, and inactive disease over time from baseline to Week 52 of the cumulative period are shown in Fig. 2. At Week 16, 18/20 (90%) patients achieved a JIA-ACR30 response (primary endpoint). JIA-ACR50, 70, and 90 response rates, and inactive disease rate were 75.0, 70.0, 35.0, and 25.0%, respectively. During the cumulative period to Week 52, JIA-ACR30 and 50 response rates increased progressively from Week 2 (first assessment) to Week 16 (end of the short-term period) and remained high to Week 52 (Fig. 2). JIA-ACR70 and 90 response rates and inactive disease rate also gradually increased to Week 16 followed by a sustained improvement to Week 52 (Fig. 2). At Week 52 (*n* = 18), the JIA-ACR30, 50, 70, and 90 response rates, and inactive disease rate were 88.9, 88.9, 83.3, 66.7, and 44.4%, respectively. In the subgroups analyses, no marked apparent differences were observed on JIA-ACR30 response rates at Week 16 regardless of sex or age at baseline, disease subtype at study entry, concomitant MTX at study Day 1 or prior biologic DMARD therapy (data not shown). However, due to the small sample size of 20 patients in this study, these data should be interpreted carefully and with caution.

**Fig. 3:**
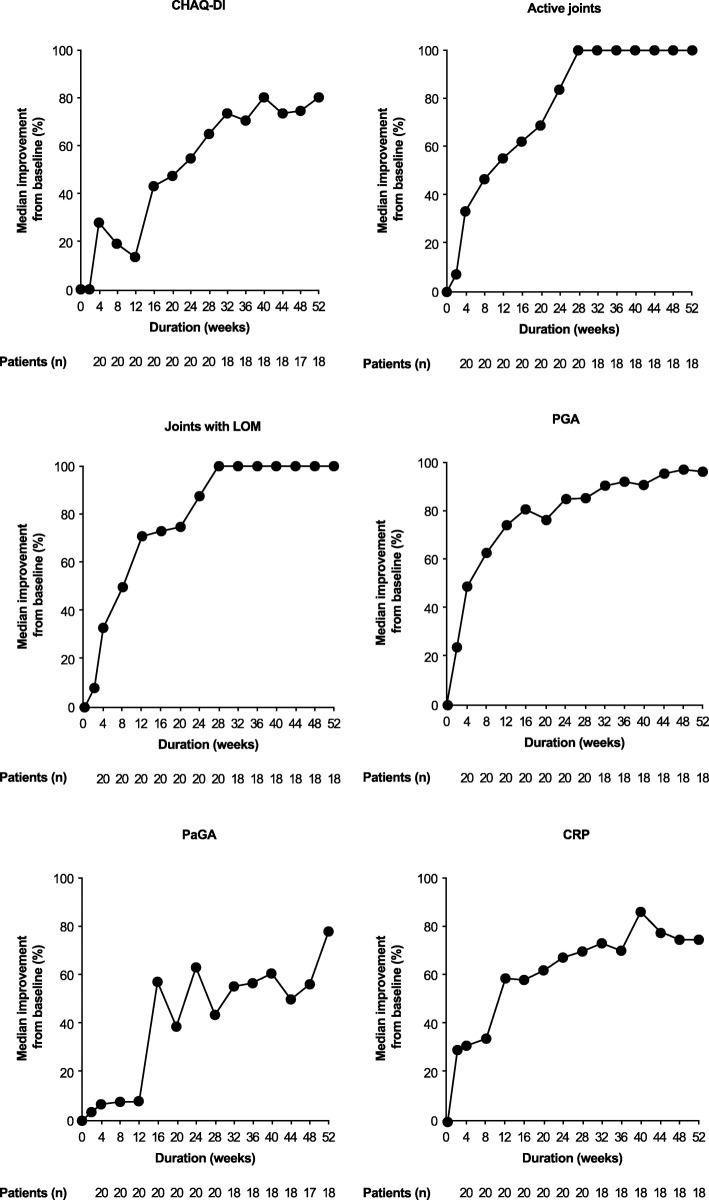
Time course of JIA-ACR core set variables improvement from baseline to Week 52 of the cumulative period. The six JIA-ACR core set variables were evaluated as the median (%) improvement from baseline at indicated time points (all treated patients; *N* = 20). *CHAQ-DI* Childhood Health Assessment Questionnaire-Disability Index, *CRP* C-reactive protein, *JIA-ACR* Juvenile idiopathic arthritis-American College of Rheumatology criteria, *LOM* limitation of motion, *PaGA* Parental Global Assessment of patient overall well-being, *PGA* Physician Global Assessment

All six JIA-ACR core set variables improved from baseline to Week 16 and throughout the cumulative period to Week 52 (Fig. 3). Rapid improvement, as early as Week 2, was observed for number of active joints, number of joints with LOM, PGA score, and CRP level. The improvement observed for active joints and joints with LOM plateaued at Week 28 but was sustained thereafter out to Week 52, whereas improvements in PGA and CRP continued to increase up to Week 52. Little, if any, improvement in CHAQ-DI or PaGA was observed during the early phase of the study (within 12 weeks of starting treatment with abatacept); however, both parameters started to show continuous improvement after 12 weeks of treatment.

**Fig. 4:**
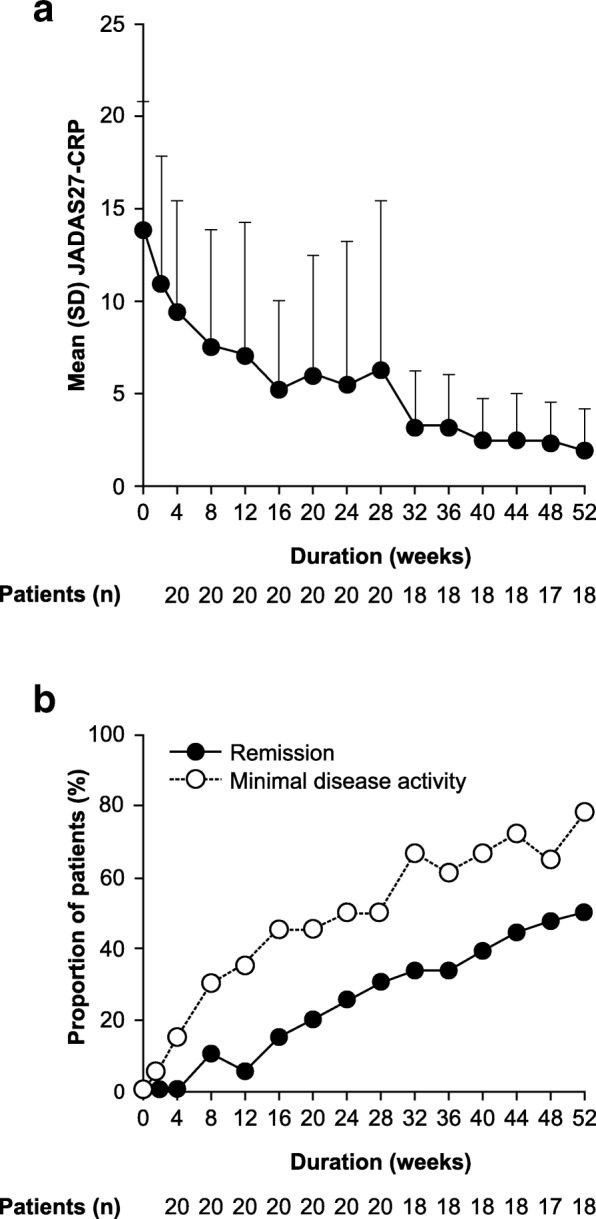
Time course of JADAS27-CRP from baseline to Week 52 of the cumulative period. **a** Mean (SD) JADAS27-CRP score and (**b**) the proportion of patients in remission (JADAS27-CRP < 1, closed circles) or with minimal disease activity (JADAS27-CRP < 3.8, open circles) from baseline to Week 52 of the cumulative period were evaluated at indicated time points (all treated patients; *N* = 20). Data were analyzed using observed cases (only patients who were in the study at the time point being evaluated). *JADAS27-CRP* juvenile arthritis disease activity score 27 active joint count-C-reactive protein, *SD* standard deviation

At baseline, mean JADAS27-CRP score was 13.9, with no patients in remission (JADAS27-CRP score < 1) or with minimal disease activity (JADAS27-CRP score < 3.8). Mean JADAS27-CRP score gradually decreased over time from baseline to Week 52 (Fig. 4a), with a mean change from baseline in JADAS27-CRP score of − 8.7 at Week 16, and –10.8 at Week 52. Remission was achieved in 3/20 (15%) patients at Week 16 and in 9/18 (50%) patients at Week 52 (Fig. 4b). Minimal disease activity was reported in 9/20 (45%) patients at Week 16 and 14/18 (78%) patients at Week 52 (Fig. 4b).

### Pharmacokinetics

**Fig. 5:**
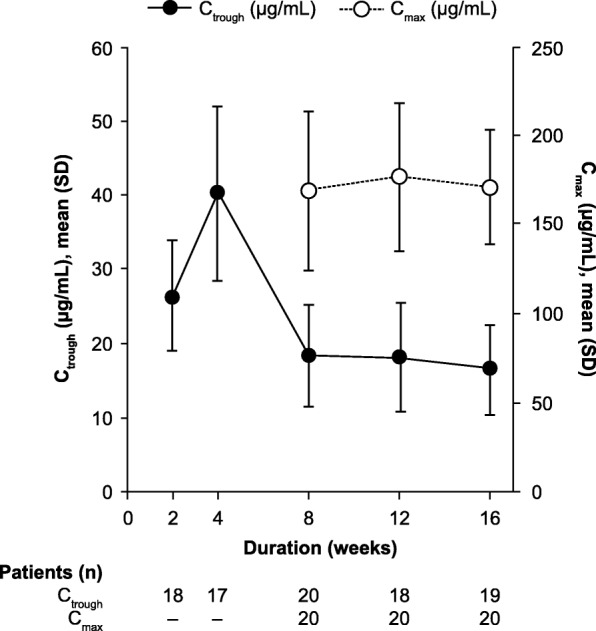
Pharmacokinetics of abatacept. Pharmacokinetics of abatacept were evaluated during the short-term period, to Week 16 at indicated time points (all treated patients; *N* = 20). Data are shown as mean (SD) for C_trough_ (closed circles, left axis) and C_max_ (open circles, right axis). *C*_*trough*_ predose observed serum concentration, *C*_*max*_ maximum observed serum concentration, *SD* standard deviation

Mean C_trough_ serum abatacept levels increased from Week 2 to Week 4, then decreased to Week 8. C_trough_ levels were maintained at 17–18 μg/mL from Week 8 for the remainder of the short-term period (Fig. 5). Mean C_max_ levels were maintained from Week 8 to 16 (Fig. 5).

### Safety

During the short-term period, all patients had at least one AE, all of which were mild or moderate in intensity; treatment-related AEs were observed in 5/20 (25%) patients (Table 2). Infections were the most commonly reported AEs in the short-term period, of which the most frequent were nasopharyngitis (*n* = 5 [25%]) and pharyngitis (*n* = 5 [25%]). All other AEs across all MedDRA preferred terms were reported in no more than two patients. Two patients experienced SAEs (gastroenteritis [mild, related to study drug] and an exacerbation of a pre-existing disease [mild, unrelated to study drug]). No patients were diagnosed with a malignancy or an autoimmune disorder, other than the aforementioned exacerbation of a pre-existing disease. There were no discontinuations due to AEs, and no deaths occurred during the short-term period.

**Table 2:** Patients with adverse events reported during the short-term period up to Week 16 and cumulative period (all treated patients; *N* = 20)

Safety events	Short-term period (Week 16)	Cumulative period
Deaths^a^	0	0
SAEs^b^	2 (10)	4 (20)
Related SAEs	1 (5)	1 (5)
Discontinued study treatment due to AEs	0	0
AEs	20 (100)	20 (100)
Related AEs	5 (25)	6 (30)
AEs of special interest		
Infections	16 (80)	20 (100)
Malignancies	0	0
Autoimmune disorders^c^	1 (5.0)	0
Infusion reactions		
Acute infusional	0	1 (5)
Peri-infusional^d^	2 (10)	5 (25)
Other AEs within 24 h^e^	4 (20)	12 (60)

The short-term period includes data up to 56 days after the last dose in the short-term period or start of the long-term period, whichever occurred first

The cumulative period includes data from the first dose in the short-term period up to 56 days post the last dose in the cumulative period

^a^Data include deaths reported during each period including those that occurred > 56 days after the last dose

^b^SAEs include hospitalizations for elective surgical procedures

^c^This event was not new onset, but worsening of the underlying disease (JIA)

^d^Defined as AEs that occurred during the first 24 hours after the start of abatacept infusion and are included in the pre-specified Medical Dictionary for Regulatory Activities list of peri-infusional events of interest

^e^Defined as AEs that occurred after the start of abatacept infusion but are not included in the list of peri-infusional AEs of special interest

*AE* adverse event, *JIA* juvenile idiopathic arthritis, *SAE* serious adverse event

During the cumulative period, all AEs were mild or moderate in intensity except for one case of severe gastroenteritis. The most common AEs were infections, of which the most frequent were nasopharyngitis (*n* = 14), pharyngitis (*n* = 10), and influenza (*n* = 7). SAEs were reported in four patients (gastroenteritis [severe, related to study drug, occurred on Days 21 and 362], varicella [mild, unrelated to study drug, occurred on Day 246], viral tonsillitis [moderate, unrelated to study drug, occurred on Day 575], and an exacerbation of a pre-existing pJIA [mild, unrelated to study drug, occurred on Day 58]). No malignancies or additional autoimmune disorders were reported during the cumulative period. There were no deaths or discontinuations due to AEs (Table 2).

### Immunogenicity

During the short-term period, none of the patients exhibited a positive signal for ADAs. During the cumulative period, one patient tested positive for antibodies to abatacept CTLA4 ± Ig, at Weeks 32 and 52, with titers of 304 and 476, respectively. This patient had JIA-ACR30, 50, and 70 but not JIA-ACR90 responses in the short-term and long-term periods. No association was observed between positive immunogenicity and loss of efficacy, safety signals, or PK in this patient.

## Discussion

In this phase III study of IV abatacept therapy in Japanese patients with pJIA and inadequate response or intolerance to biologic DMARDs or MTX therapy, the primary endpoint of JIA-ACR30 response at Week 16 was achieved, with 90% of patients responding. For a sample size of 20 patients, a JIA-ACR30 response rate of 65% (two-sided exact 95% CI of 40.8 to 84.6%) was predicted based on a previous phase III study of IV abatacept in patients with active pJIA (NCT00095173) [26]. In the current study, IV abatacept had a beneficial effect on physical function, as demonstrated by improvements in CHAQ-DI relative to baseline measurements, and was well tolerated over the 52-week cumulative period.

In addition to meeting the primary efficacy endpoint (JIA-ACR30 response), JIA-ACR50 and 70 responses were also observed as early as Week 2 and response rates continued to increase through to Week 16, plateauing around Week 36. The rates of more stringent JIA-ACR response measures and inactive disease status increased steadily over time with continued abatacept treatment during the cumulative period, similar to the kinetics observed for improvement in JIA-ACR30 response rates. In the international, multicenter, phase III clinical trial and real-world study of IV abatacept, sustained increase in the JIA-ACR response rates during abatacept treatment was also demonstrated. However, in this study, the JIA-ACR30, 50, 70 and 90 response rates, and inactive disease status at week 16 were numerically higher than the corresponding response rates observed in the international trial (65, 50, 28, 13 and 13%, respectively) [26]. Importantly, in this study, the patients may have had an early disease onset, with numerically lower disease duration, number of active joints and CHAQ-DI at baseline than those reported in the international study, which may support the notion of a potential effect of patient background, specifically race and ethnicity, on response to abatacept treatment and emphasize the importance of establishing the efficacy and safety profile of the drug in specific patient populations. In addition, the JIA-ACR90 response of 66.7% at Week 52 observed in this study was similar to that observed in a study of the IL-6 receptor inhibitor tocilizumab in pJIA, in which JIA-ACR90 response was 64.7% at Week 48 [40].

The present findings are also consistent with those from previous studies that assessed the efficacy of abatacept based on JIA-ACR30 response in different JIA subtypes (extended oligoarticular JIA and RF-positive and RF-negative polyarticular JIA) [12, 15, 19, 41]. In subgroup efficacy analyses, there were no marked differences in JIA-ACR30 response rates regardless of sex or age at study baseline, JIA subtype, concomitant MTX dose, or prior biologic therapy. However, it should be noted that statistical difference was not formally analyzed in these subgroups and limited conclusions can be drawn due to the very small sample sizes. JIA-ACR30 response rates achieved with abatacept have previously been found to be unaffected by JIA subtype, but may be lower in patients who have received prior anti-TNF therapy compared with those who were anti-TNF naïve at abatacept initiation [26]. The improvement from baseline in each of the six JIA-ACR core set variables during the cumulative period supports the increased overall JIA-ACR response rates. Gradual and continuous improvements were seen in several measures of disease activity, including CHAQ-DI scores, suggesting that abatacept treatment is associated with a reduction in physical disability. These results concur with previous reports of abatacept treatment in patients with pJIA from outside Japan [15, 26]. Interestingly, in this study, improvement in PaGA was slower and of a lower magnitude than that in PGA over time, which may be a reflection of parents having a worse perception of their child’s condition, or higher expectations of a new investigational drug, than the treating physician. These findings, suggesting that parents might overestimate a child’s condition, are somewhat consistent with those from a previous study in which children with JIA reported that their health-related quality of life was better than that reported by their parents [42]. It should be noted that PGA, which was evaluated by objective parameters such as joint symptoms and CRP, promptly improved, implying that it might not be appropriate to make a decision for insufficiency of the therapeutic intervention based on only PaGA and CHAQ (although the decision is usually made 3 months after starting the treatment). It should be taken into account that PaGA and PGA measure different aspects of disease and should not be considered redundant.

JADAS has been recognized recently as a valuable measure of disease activity within the clinical trials and routine practice, as it allows for a more accurate determination of the disease activity state than JIA-ACR response criteria [43]. Mean score and change from baseline in JADAS27-CRP, as well as proportion of patients in JADAS27-CRP-defined remission and with minimal disease activity, exploratory endpoints in this trial, were investigated to determine the disease activity state of patients with pJIA over the study period. [43] In this study, proportion of patients with JADAS27-CRP-defined remission (JADAS27-CRP score < 1) and with minimal disease activity (JADAS27-CRP score < 3.8) increased over time with abatacept treatment. Importantly, the mean change from baseline in JADAS27-CRP score decreased early and remained low over time with abatacept therapy, reflecting the rapid and sustained response to abatacept in this patient population.

A PK steady state was achieved by Week 8, with C_trough_ levels maintained above the target therapeutic level of 10 μg/mL, which has been associated with near maximal efficacy based on JIA-ACR30 responses in other pJIA patient populations [44]. The mean C_trough_ was similar to that reported in a previous study of abatacept in Japanese patients with RA [45]. Since the efficacy and PK data presented here demonstrate a gradual cumulative benefit with continuous abatacept treatment, it is possible that continuing treatment —assuming that it is well tolerated — even if initially there is minimal or no benefit, may achieve a clinical response over time.

IV abatacept was well tolerated and no new safety signals were identified. The AE profile included events of special interest (infections, malignancies, autoimmune disorders, and infusion reactions) and was comparable with that reported previously for abatacept in patients with pJIA [12, 15, 26], indicating that the safety profile does not differ markedly between Japanese patients and other populations.

No ADAs were detected during the short-term period and only one patient had a positive immunogenic response during the cumulative period. This positive response did not appear to be associated with disease flare, SAEs, acute infusional AEs, hypersensitivity, autoimmune disorders, or low abatacept serum concentrations; importantly, JIA-ACR30, 50, and 70 responses were achieved in this patient. These results are consistent with previous reports of patients with pJIA testing positive for ADAs where no effects on efficacy, safety, or PK were observed [12].

Limitations to this study should be noted. First, the small sample size should be considered when interpreting the results. Furthermore, this was an open-label, single-arm study, without a placebo or other active treatment arm for comparison of the efficacy and safety of abatacept in this Japanese patient population. However, the findings presented here are consistent with those from other studies in JIA and with the large body of abatacept clinical data available for adult patients with RA. Further studies of randomized design and on large numbers of patients may be warranted to support the results observed in this trial.

## Conclusions

In this study of Japanese children and adolescents with pJIA, IV abatacept treatment was effective, with benefits accruing over time, and no new safety concerns were identified. These data suggest that IV abatacept treatment provides an effective and well-tolerated treatment option for Japanese patients with active pJIA who do not tolerate existing first-line treatment.

## Abbreviations

ACR: American College of Rheumatology
ACR: American College of Rheumatology
ADA: Anti-drug antibodies
AE: Adverse event
CHAQ-DI: Childhood Health Assessment Questionnaire-Disability Index
CIs: Confidence intervals
CRP: C-reactive protein
CTLA4: Cytotoxic T-lymphocyte-associated protein-4
DMARD: Disease-modifying anti-rheumatic drug
ICORE: Institute of Clinical Outcomes Research and Education
Ig: Immunoglobulin
IL: Interleukin
IV: Intravenous
JADAS27-CRP: Juvenile arthritis disease activity score 27 active joint count-CRP
JADAS27-CRP: Juvenile Arthritis Disease Activity Score in 27 joints using C-reactive protein
JIA: Juvenile idiopathic arthritis
JIA-ACR30: JIA-American College of Rheumatology criteria 30
LOM: Limitation of motion
MDA: Minimal disease activity
MedDRA: Medical Dictionary for Regulatory Activities version
MTX: Methotrexate
PaGA: Parental Global Assessment
pJIA: Polyarticular-course juvenile idiopathic arthritis
PK: Pharmacokinetics
RA: Rheumatoid arthritis
RF: Rheumatoid factor
SAEs: Serious AEs
TNFi: Tumor necrosis factor-α inhibitor
